# Study on the influence mechanism of adoption of smart agriculture technology behavior

**DOI:** 10.1038/s41598-023-35091-x

**Published:** 2023-05-26

**Authors:** Jingjin Li, Guoyong Liu, Yulan Chen, Rongyao Li

**Affiliations:** College of Economics and Trade, Xinjiang Agricultural University, Ürümqi, Xinjiang China

**Keywords:** Psychology, Environmental sciences, Environmental social sciences

## Abstract

Smart agricultural (SA) technology has become a technological support for modern agriculture. By exploring the decision-making process and psychological motivation of farmers in adopting SA technology, it is conducive to achieving the popularisation of SA technology and promoting the modernisation of agriculture. Based on microscopic research data, a Structural Equation Model (SEM) is used to analyse the influencing factors and extent of cotton farmers’ adoption of SA technologies, using Deconstructive Theory of Planned Behavior (DTPB) as the analytical framework. This was combined with in-depth interviews to further reveal the motivations and influencing mechanisms of cotton farmers’ adoption of SA technologies. The results show that under the behavioural belief dimension, cotton farmers value the positive effect of perceived usefulness even though the risk of the technology itself has a dampening effect on adoption intentions. Under the normative belief dimension, superior influence influenced the willingness to adopt SA technologies to a greater extent than peer influence. Under the control belief dimension, factors such as self-efficacy and information channels influence willingness to adopt technology and behaviour. In addition, behavioural attitudes, subjective norms, and perceived behavioural control all contribute to cotton farmers’ willingness to adopt SA technologies, and can also influence behaviour directly or indirectly through willingness to adopt. Policy and technology satisfaction positively moderate the transition from willingness to behaviour. Therefore, preferential policies are proposed to reduce the cost of adopting SA technologies; to continuously improve the level of SA technologies; to establish SA technology test plots to provide a reference base; and to increase knowledge training on SA and expand access to information.

## Introduction

Smart agriculture (SA) relies on various SA technologies, which have been commonly applied in the field of agriculture^1–3^. SA has become a global trend in the development of agricultural modernization^4^, with developed countries using high-level agricultural technology and agricultural subsidy support as a carrier^3^ and capital flows and market expansion as a backing, objectively forming a squeeze and control on the development of agricultural industries in developing countries. Therefore, for more than a decade in a row, China has had relevant documents dealing with SA, which is an important initiative to break through the current bottleneck of traditional agricultural development^5^, achieve high-quality, high-efficiency and sustainable development of agriculture^6,7^, and take the lead in modernising agriculture and rural areas.

The current problem of ageing farmers remains acute^8^, which can significantly weaken the level of human capital in agriculture, severely limit the scope for technology diffusion and become a potential threat to the technological transformation of agriculture^9^. In the reality of high production costs and risks, there is also a need to improve production efficiency through SA technologies. Secondly, with the rise in cotton prices and subsidy policies, cotton farmers have been stimulated to demand higher standards of technology. In addition, current agricultural production suffers from inefficient use of arable land and excessive use of agricultural resources^10,11^. At the same time, excessive inorganic inputs have caused a decline in the quality of agricultural products, a decline in land strength, and pollution from agricultural surface sources. Such a reality indicates that there is an urgent need to further liberate labour, improve productivity and conserve resources, and increase farmers’ incomes and modern skills, supported by SA technologies^12^, in order to enhance endogenous rural development dynamics. Farmers, as implementers, have a direct impact on their adoption behaviour.

The concept of SA can be traced back to the “Smart Planet” concept introduced by IBM in 2009. SA is the use of SA technology as a medium for agricultural production. SA technology is led by a new generation of information technology, with intelligent information technology such as intelligent sensors, the Internet of Things and big data integrated with agriculture^13–15^, with technical characteristics such as agricultural information perception, quantitative decision-making, intelligent control management, accurate measurement and personalised services. Current research on SA mainly includes the definition and characteristics of the concept of SA, the development status of SA, problems and countermeasures^16–18^. Scholars have explored the core technologies inSA in depth, mainly focusing on applications in agricultural production as well as operation and management^19^, as well as typical development models and practices of SA at home and abroad^20^.

Based on the classical “economic man hypothesis”, most scholars regard farmers as rational economic men and believe that farmers’ technology adoption behavioural decisions are rational and economic^21,22^. However, farmers’ behaviour deviates from economic rationality due to their personal characteristics and subjective biases in the decision-making process of behavioural response. Therefore, scholars have conducted research from different perspectives. The research shows that farmers’ behavioural decisions are influenced by a combination of internal and external factors, which leads to an in-depth exploration of the logic of farmers’ behaviour. Farmers’ behavioural preferences vary by gender and age^23–25^, which is a root factor for the differences in farmers’ perceptions^26–28^. Behaviour is also influenced by a combination of other individual characteristics such as literacy, part-time employment, access to information, risk perception, social networks and other household characteristics^29–31^. Resource endowment constraints, in the form of land, labour and capital, are one of the key factors influencing the technology adoption behaviour of farm households^19,32–34^. The larger the size of the land, the more efficient production can be achieved when new technologies are adopted, and the more motivated farmers will be to adopt them^35,36^. Smallholder farmers are constrained by resources, and agricultural social services can effectively mitigate technological barriers and promote farmer technology adoption^37,38^. External factors revolve around the policy environment and risk appetite. Government subsidies and policy regulation are the main instruments adopted by the government. Subsidy policies provide incentives for farmers to adopt appropriate technologies according to their farming needs^39,40^, while institutional constraints discourage adoption behaviour^15^. The government also motivates farmers through agricultural technology training and advocacy guidance^41–43^. In addition, agriculture is naturally weak and exposed to multiple risks from natural disasters, market changes and other shocks^44^. When adopting a new technology, farmers are bound to fully consider the possible risk factors and thus allocate their existing capital efficiently.

The research at this stage has been fruitful, but there is still room for expansion, and possible innovations for this paper exist in the following areas:

1. Expansion of research objects. Most of the literature on green, low-carbon and climate-related agricultural technologies is lacking in the scientific analysis and precise pulse on the use of SA technologies. This paper provides an in-depth analysis of the behavioural logic of cotton farmers’ adoption of SA technologies from a micro perspective.
2. Innovation in research theory and methodology. Most scholars’ research on farmers’ technology adoption has mainly focused on the Theory of Planned Behaviour (TPB)^45–47^. However, the factors selected for the indicators of this theory are too single, and the research methods mostly adopt models such as Logistic^21^ and Probit^31^. This paper takes farmers’ subjective feelings as the reference point, incorporates psychological factors such as cognitive state and perceived risk into the Deconstructive Theory of Planned Behaviour (DTPB), and uses Structural Equation Modelling (SEM) to explore the underlying mechanisms and pathways of each influencing factor in depth.
3. Additions and refinements to the research mechanism. Further test whether there is a mediating effect of willingness to adopt technology in each of the three belief dimensions and behaviour? Do cotton farmers’ policy satisfaction with SA technologies and satisfaction with technology use and services play a moderating effect in the conversion of technology adoption intentions into behaviour? Furthermore, current research on technology choice is more general and there is still a proportion of cotton farmers who are unwilling or fail to adopt new technologies and what is the reason for this?

In view of this, this paper uses survey data from cotton farmers in Xinjiang to screen five SA technologies. A combination of DTPB and SEM was used to explore the impact pathways and transmission mechanisms of cotton farmers’ adoption of SA technologies. Finally, the findings of the study are summarised and corresponding countermeasures are suggested. It is hoped that this study will provide a reference for the effective adoption of SA technologies by farmers and provide new ideas for research on SA.

## Research theory and hypothesis

**Figure 1:**
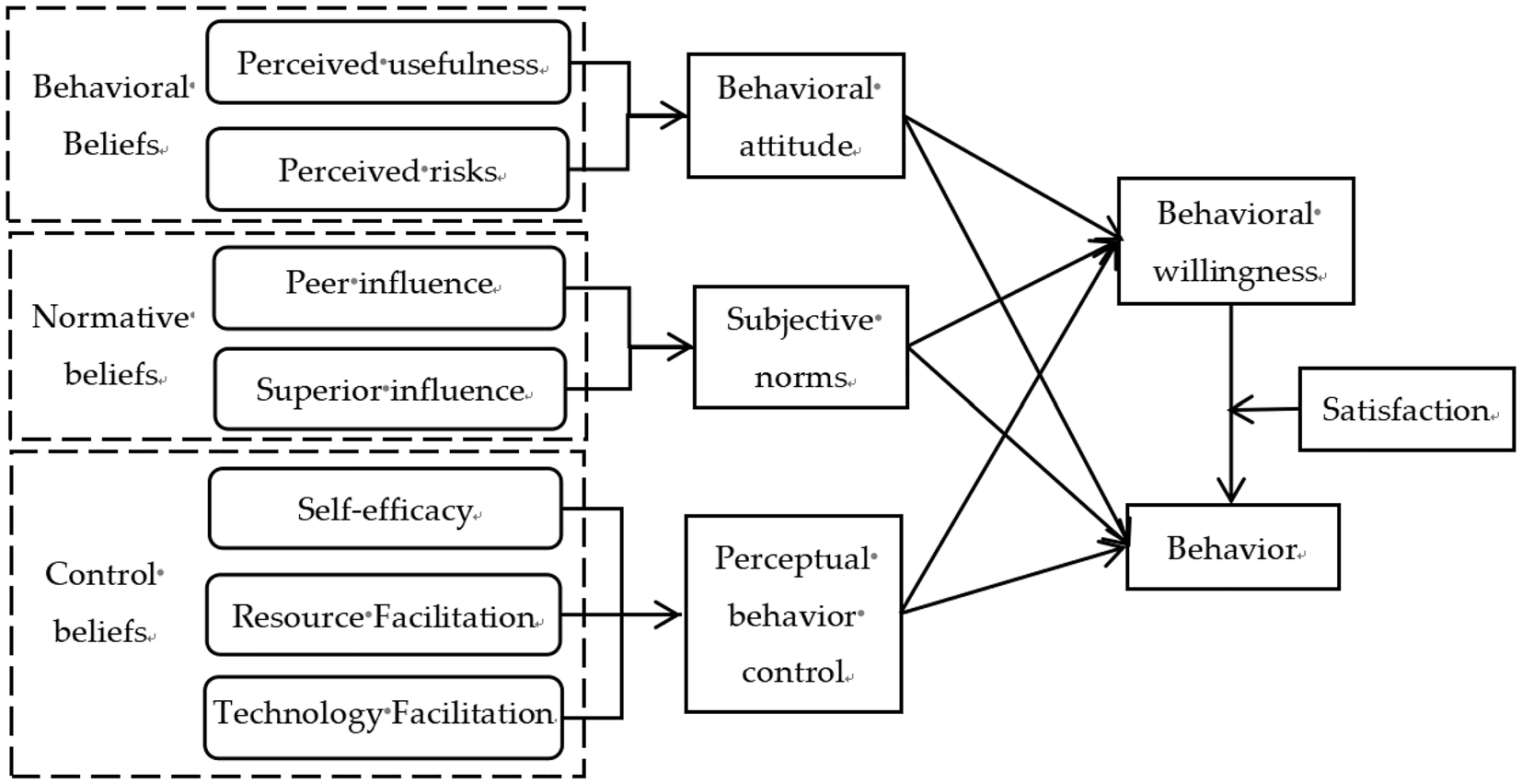
Mechanism of cotton farmers’ adoption of SA technology behavior.

The TPB theory was re-integrated and refined by multi-dimensional decomposition again to form the DTPB. the DTPB theory possesses reliability and rationality, with a stronger structure and more precise estimating power, and is more suitable for different categories of research^48^. It also investigates the deeper psychological perceptual elements of individual behaviour. Based on this, this paper draws on this theoretical model to delineate the psychological factors underlying cotton farmers’ adoption of SA technologies at a deeper level across three scales: behavioural, normative and control beliefs. In addition, policy and technology satisfaction are added as moderating variables to explore the moderating effects in the transformation of willingness to adopt technology into behaviour (Fig. 1).

Behavioural beliefs refer to cotton farmers’ positive or negative attitudes towards the adoption of SA technologies and are determinants of attitudes. In adopting new technologies, cotton farmers consider the immediate benefits and the long-term future development that they will bring^49^. In addition, the main concern of cotton farmers is that gaining benefits and risks are in conflict with each other. Not only does the adoption of new technologies bring benefits, but there is also risk taking and avoidance. As technology implementation is subject to a variety of impacts, it results in risky decisions under conditions of technological uncertainty of adoption. The more cotton farmers perceive the technology, the more positively they evaluate its convenience and effectiveness, the stronger their willingness to adopt it. Behavioural beliefs are formed when cotton farmers perceive that SA technologies are driving or hindering change. In this paper, behavioural beliefs are decomposed into two antecedent variables: perceived usefulness and perceived risk. When the benefits carried by SA adoption are higher, cotton farmers’ attitudes towards SA adoption will be higher, thus promoting behavioural intentions and behaviours; at the same time, when the risks carried by SA adoption are lower, cotton farmers’ attitudes will be more proactive, thus increasing technology adoption intentions and behaviours.

Normative beliefs refer to the external pressure cotton farmers feel to adopt and reflect the influence of institutions, organisations or systems on individual decisions. If a cotton farmer is uncertain about the outcome of a SA technique, he or she may choose to listen to the opinions of others in order to judge his or her own behaviour. And they can feel the insights or ideas relayed to them by nearby family members, friends and relatives. But cotton farmers are not only attached to a network of social relations at the level of their peers, they also rely on a network of social relations at the level of their superiors. In social group interactions, cotton farmers often deal with villages or regiments and agricultural dealers, and government promotion efforts have an impact on the probability of technology adoption and the degree of adoption by cotton farmers^17^. The government has made cotton farmers more enthusiastic about adopting new technologies through unified learning by heavily promoting SA technology and organising training on the technology^18^. At the same time, the price subsidy policy granted by the government will stimulate cotton farmers’ willingness to adopt the technology. In this paper, peer influence is split into family and close friends. The promotion efforts, publicity and subsidy mechanism of the village or regiment and local government are screened as indicators of superior influence. When social groups and superior relationships motivate cotton farmers to adopt, the greater the willingness and behaviour of cotton farmers to adopt.

Control beliefs refer to the degree of difficulty and mastery cotton farmers perceive in adopting SA technology. It covers both self and external dimensions: firstly, the cotton farmers’ perception of their own capabilities, and the formation of willingness also varies according to self-tolerance^50^. Secondly, external influences on one’s own behaviour; external forces refer to the extent to which the relevant resources at the cotton farmer’s disposal facilitate adoption. The behavioural choices of cotton farmers depend on their subjective intentions. The stronger the subjective will of the cotton farmers, the more they will take the initiative to obtain information in various ways and thus adopt it more quickly. In addition to being available in villages or missions, there are also online sources that can be accessed^51^. For example, tools such as the government’s online agricultural extension service platform, the ShakeOut platform or public numbers can be used to provide online training, browse agricultural policies or explain agricultural knowledge directly through mobile phones^52,53^. Broadening cotton farmers’ access to information channels, enhancing the timeliness and effectiveness of access to information content, grasping the latest agricultural technology adoption dynamics, narrowing the digital information gap, reducing information acquisition costs, facilitating timely agricultural decision-making by cotton farmers, and ultimately enhancing technology choices^54^. At the same time, the more resources cotton farmers believe they have and the less hindered they are, the higher their willingness to adopt. We split control beliefs into three antecedent variables of self-efficacy, resources and technology facilitation, and selected indicators of autonomous decision-making power, mastery, risk and time cost tolerance, smooth flow of information in village groups, and online information platforms. When cotton farmers have a higher degree of recognition of their own abilities and believe that they can afford the process and outcome of adoption, the stronger their willingness and behaviour to adopt will be; at the same time, when the facilitation conditions are more favourable, the more they can promote the adoption of SA technology.

As individual SA technologies are not yet widespread and there is an information asymmetry, cotton farmers do not necessarily translate into behaviour even if they have the will to adopt SA technologies. Government policy, as an important tool and measure of national macro-control, is an enabler of transformative production and development in China’s agriculture. The behaviour of cotton farmers in adopting SA technologies has positive externalities and requires the support and guidance of government policies, and the satisfaction of cotton farmers with these policies will affect the transformation of willingness into actual behaviour. In addition, when friends, relatives, demonstration households and other growers adopt SA technologies and achieve benefits, the demonstration effect and imitation effect will be generated, and the satisfaction of cotton farmers with SA technologies will enhance the change from willingness to behavioural adoption. Therefore, this paper introduces policy and technical service and effect satisfaction as moderating variables to investigate their moderating effects on behavioural willingness to adopt SA technology.

## Methodology

### Data collection

China is the world’s largest cotton producer, and Xinjiang is the main cotton-producing region in China. The methodology involved in the research was carried out in accordance with the guidelines and regulations of the Humanities and Social Sciences Research Committee of Xinjiang Agricultural University in China. The research proposal was approved by this committee and the university issued a research letter. The survey was conducted from July to September 2022. The research was conducted in advance of the research area, and then communication was made with the local agricultural authorities in advance to determine the exact timing of the research. After obtaining verbal informed consent from the farmers prior to the research, the local agricultural department provided the researchers with basic information about the farmers. To ensure the representativeness and diversity of the data, the survey selected cotton farmers of different types and business sizes from the major cotton-producing regions in northern Xinjiang: Changji County, Hutubi County and Manas County in Changji Prefecture, Bole City in Bo Prefecture and the 6th and 8th Divisions of the Xinjiang Production and Construction Corps for questionnaire surveys and household interviews. A total of 400 questionnaires were distributed and 394 valid questionnaires were collated, with an effective rate of 98.5%.

### Measurement

Due to the small application range of some SA technologies, in order to guarantee the accuracy of the data. The assignment of one variable for the SA technology used in the study was scored according to whether five technologies were used: GPS positioning system sowing integrated machine, water and fertiliser integrated intelligent drip irrigation system, Unmanned aircraft, cotton field environmental monitoring and control system, and Beidou navigation system for baled cotton picking integrated machine. SEM and mediated effects were run using AMOS 28.0 software. Moderating effects were then analysed using SPSS 28.0 software, with moderating variables selected to rate cotton farmers’ satisfaction with the policy and technology use effects and services of the five SA technologies, and finally averaged. All other variables were assigned using a five-point Likert scale. Where 1—completely disagree, 2—not very much agree, 3—average, 4—basically agree and 5—completely agree. The survey had a total of 38 question items and 14 latent variables (Table 1).

**Table 1:** Variable setting and reliability validity testing.

Variable	Code	Observed variable	AVE	Std.
Perceived usefulnes	PU1	SA technology can increase economic returns	3.596	0.788
	PU2	SA technology can increase yield	3.556	0.824
	PU3	SA technology can benefit the cotton industry	3.307	1.288
	PU4	SA technology can reduce labor input and save energy and physical strength	3.249	1.248
	PU5	SA technology can reduce water consumption, pesticide and fertilizer use	3.406	0.851
Perceived risks	PR1	The adoption of SA technology is ineffective and there are risks of immature technology	2.282	0.777
	PR2	SA technology can increase costs	2.751	1.075
	PR3	Lack of follow-up guidance or maintenance by technical staff after using SA technology	2.409	0.789
Peer influence	PI1	My family supports me to adopt SA technology	3.594	0.933
	PI2	My family supports me to adopt SA technology	3.693	0.977
Superiors influence	SI1	Villages/crops support the adoption of SA technology	3.188	1.008
	SI2	Agricultural distributors promote the adoption of SA technology	3.305	1.119
	SI3	Government policies to promote SA technology	3.561	1.125
	SI4	Government subsidies for the acquisition of SA machines	3.371	1.076
Self-efficacy	SE1	It is up to me to decide whether to adopt SA technology or not	3.566	1.214
	SE2	I am capable of learning and mastering SA technology	3.497	0.758
	SE3	I can bear the risks associated with adopting SA technology	3.340	1.104
Resource facilitation	RF1	My family has enough financial support to invest in SA technology	3.046	0.866
	RF2	I have enough time to learn SA technology	3.609	0.843
Technology facilitation	EF1	Information channels for SA technology are available in the village/crops	3.584	0.770
	EF2	Web-based information platform to promote SA technology	3.622	0.791
Behavioral attitude	BA1	I think the advantages of SA technology outweigh the disadvantages	3.553	1.150
	BA2	I think it is necessary to adopt SA technology	3.893	0.808
	BA3	I think SA technology is a future development trend	3.891	1.261
Subjective norms	SN1	People who have influence on my family agree with the adoption of SA technology in my family	3.655	0.970
	SN2	Relevant systems and guarantees support the adoption of SA technology in my household	3.761	0.934
Perceptual behavior control	PBC1	I have the conditions to adopt SA technology	3.538	0.709
	PBC2	I have easy access to information about SA technology	3.734	0.766
	PBC3	I can easily adopt SA technology	3.505	0.788
Behavioral willingness	BW1	Willing to adopt SA technology	3.746	1.111
	BW2	Willing to attend training on SA technology	3.754	1.202
	BW3	Willing to recommend people around me to use SA technology	3.718	1.175
	BW4	Willing to invest time, money and adopt SA technology	3.406	0.854
Behavior	B1	Adoption of SA technologies	3.708	0.776
	B2	Level of knowledge about SA technologies	3.431	0.816
	B3	Proactive participation in training on SA technology	3.622	0.896
Technology satisfaction	TS	Satisfaction with the effectiveness of using SA technologies and services	3.182	1.184
Policy satisfaction	PS	Satisfaction with policy onSA technology	3.063	1.229

## Results

**Figure 2:**
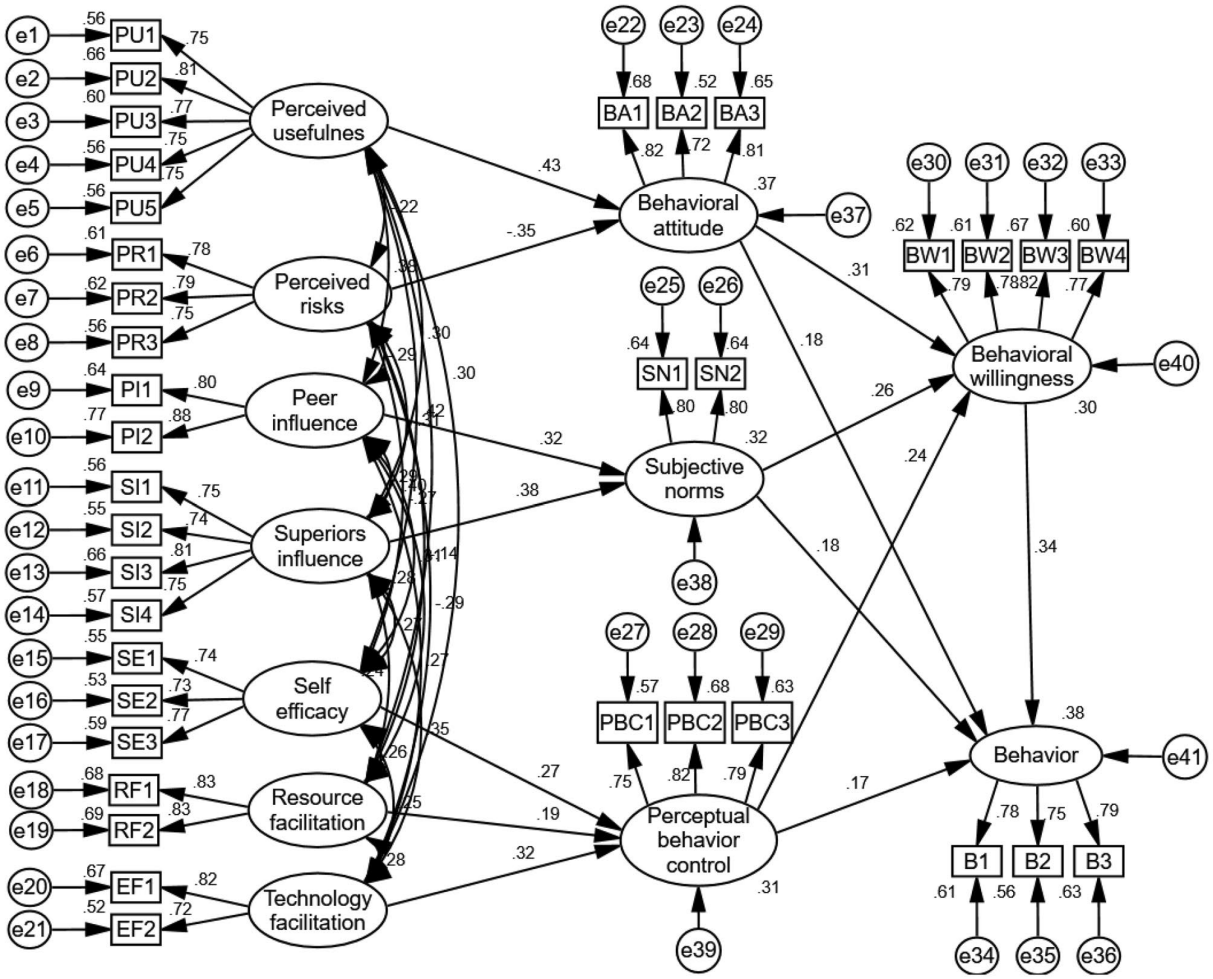
Structural equation model and path coefficients.

Behavioural attitudes have a significant influence on cotton farmers’ willingness to adopt SA technologies, followed by subjective norms and perceived behavioural control. Furthermore, the greatest degree of influence on cotton farmers’ adoption of SA technology was behavioural willingness and the least was perceived behavioural control. The path coefficient of perceived usefulness to behavioural attitudes in behavioural beliefs was 0.426, which was higher than the path coefficient of perceived risk − 0.353. This indicates that in adopting SA technology, cotton farmers are more concerned about the important role of changes in welfare levels brought about by SA technology itself, even though the presence of partial risk inhibits their intention to adopt SA technology. The standardised path coefficients of superior influence and peer influence on subjective norms in the normative beliefs were 0.385 and 0.316 respectively, indicating that cotton farmers are more dependent on their superiors in the decision-making process. The path coefficients of self-efficacy, resources and technological facilitation on perceived behavioural control in control beliefs were 0.27, 0.185 and 0.315, respectively, indicating that technological facilitation had a stronger influence on cotton farmers (Fig. 2).

### Mediating effects

In order to further verify the mechanism of the role played by cotton farmers’ willingness to adopt SA technology, the Bootstrap method was used to analyse the impact of the mediating effect under different paths. Table 2 shows that the corresponding confidence intervals for each path do not include 0, indicating that the mediating effects of the different paths are significant and all have a partial mediating effect. The standardised direct effects for the three different paths between behavioural attitudes, subjective norms, perceived behavioural control, willingness to adopt and behaviour were 0.184, 0.184 and 0.171 respectively, the indirect effects were 0.105, 0.089 and 0.081 respectively, and the total effects were 0.288, 0.273 and 0.252 respectively.

**Table 2:** Results of mediating effect test (bootstrap = 5000).

Path	Effect		SE	Bias corrected (95%)			Percentile method (95%)		
				LLCI	ULCI	P	LLCI	ULCI	P
Behavioral attitude → Behavioral willingness → Behavior	Direct effect	0.184	0.066	0.054	0.314	0.006	0.054	0.314	0.006
	Indirect effect	0.105	0.029	0.056	0.171	0.000	0.053	0.168	0.000
	Total effect	0.288	0.064	0.162	0.412	0.000	0.162	0.412	0.000
Subjective norms → Behavioral willingness → Behavior	Direct effect	0.184	0.059	0.074	0.300	0.001	0.068	0.296	0.002
	Indirect effect	0.089	0.028	0.044	0.152	0.000	0.042	0.150	0.000
	Total effect	0.273	0.058	0.160	0.387	0.000	0.160	0.387	0.000
Perceptual behavior control → Behavioral willingness → Behavior	Direct effect	0.171	0.062	0.044	0.288	0.008	0.046	0.289	0.007
	Indirect effect	0.081	0.024	0.043	0.139	0.000	0.037	0.132	0.000
	Total effect	0.252	0.064	0.122	0.375	0.000	0.122	0.374	0.000

### Moderating effects

Cotton farmers’ willingness to adopt technology influences behaviour, and the degree of influence is moderated by satisfaction with policy and satisfaction with technology services and outcomes. The interaction between adoption intentions and satisfaction with policy and technical services and effects had a significant effect on adoption behaviour, and the path coefficient was positive, indicating that policy satisfaction and satisfaction with technical services and effects positively moderated the effect of adoption intentions on adoption behaviour (Table 3).

**Table 3:** Results of analysis of moderation effects.

	Behavior		Behavior	
Behavioral willingness	0.426	9.692	0.454	11.046
Technology satisfaction	0.233	5.31	0.223	5.451
Interaction item 1			0.314	7.807
R2	0.275		0.373	
F	74.209***		77.374***	
Behavioral willingness	0.446	10.161	0.47	10.947
Policy satisfaction	0.197	4.495	0.191	4.477
Interaction item 2			0.206	4.849
R2	0.261		0.303	
F	69.062***		56.529***	

***p < 0.001.

## Discussion

The observed variables under each belief dimension have heterogeneous effects on willingness and behaviour to adopt smart farming technologies. This result has been confirmed by scholars. Among them, behavioural attitudes, subjective norms and perceived behavioural control play a positive role on willingness^45–47^. This paper also finds that willingness to adopt technology positively promotes technology adoption behaviour.

### Behavioural beliefs dimension

According to the ‘economic man’ hypothesis, the ability of a new technology to secure or enhance economic benefits is a major consideration in the adoption of SA technology. Other studies have also shown that the income and benefits of farming have an impact on the willingness of farmers to engage in farming^55,56^. Although returns are highly attractive, the impact of the technology on returns is much smaller than the impact of the price of cotton. Secondly, the effect on increasing yields and reducing farming costs is less obvious, as yields are mainly influenced by factors such as weather and seed. Again, the SA technology reduces material inputs through accurate measurement, leading to lower production costs. In addition, the reduction in labour plays an integral role in the willingness to adopt SA technologies. As a result of urbanisation, there has been a significant labour exodus from the countryside, leading to an ageing rural population at this stage^8^, and cotton farmers are more interested in adopting SA technologies to save time and reduce their workload. The benefits of technology come with risks. This paper is consistent with other scholars who have concluded that there is an inhibitory effect of risk perception on behavioural intentions^57^. Cotton farmers are often reluctant to adopt SA technology and related services if they perceive that the results are not good and that they will not only not benefit from the technology but will also suffer financial losses. In addition, the SA technology itself and the lack of technical staff to follow up and guide the adoption or the failure of machinery to be repaired in time lead to a poor perception of use by cotton farmers, who after a short period of implementation will still be planting in the traditional way as before.

### Normative beliefs dimension

With the government and agronomy-related departments promoting smart agriculture policies through top-down efforts, some localities have established national agricultural science and technology parks to provide technical guidance to cotton farmers in the field, playing a positive demonstration and driving role. These technology parks can facilitate the diffusion and adoption of technology and rapid market penetration^58^. It enables cotton farmers to understand more directly the functions and benefits of SA technology, thus eliminating their concerns about SA technology. The dealers mainly promote agricultural products and do not promote the new technology to a great extent. Secondly, some villages, regimental collectives or new management bodies and other organisations will force cotton farmers to adopt a particular SA technology, and this mode of promotion causes a disconnect between the supply of technology and actual demand. However, this collective technical service effectively alleviates the high cost, high risk and lack of technical management capacity faced by cotton farmers in adopting SA technologies through unified production and technical management. In addition, the government subsidises the purchase of SA machinery. However, the subsidies are only for new domestic machinery, while second-hand and imported machinery are not eligible. Numerous studies have demonstrated the need for good access to information^59^. Moreover, policy incentives can sustain and scale the adoption of new technologies by farmers^41–43,60^. The connection of social network relations formed by cotton farmers based on geography and kinship makes information transfer and collective communication and decision making the main way of technology diffusion^61^. In rural societies, cotton farmers learn from the effects and experiences of ‘opinion leaders’, such as local demonstrators, neighbours and co-operative members, who prioritise the adoption of new technologies, and then emulate them to reduce the risks and uncertainties of adopting SA technologies. In addition, the vast majority of cotton farmers make decisions with the input of family members. However, some family members are not involved in growing cotton and cannot provide constructive advice to inform their decisions. Therefore, peer and superior social networks can have an impact on behaviour^62^.

### Control beliefs dimension

From the point of view of the cotton farmers’ own risk tolerance. Most of the risk-averse farmers are large growers or young, well-educated cotton farmers who are willing to try out new technologies as a priority. This result is further evidence of the more positive attitude of younger farmers^63,64^. Large growers with test plots choose a small portion of their land for experimentation. Risk-averse people, on the other hand, are generally small or older cotton farmers, who are resistant to adopting new technologies and less risk-averse. They are relatively experienced in growing and are more satisfied with the use of current technology and less receptive to new technology^65^. At this stage, the traditional, conservative, smallholder mindset that refuses to accept new things is still prevalent. In terms of finance, cotton farmers will work off funds through agricultural loans and other means, even if they do not have sufficient funds, as long as they see the benefits of the technology. Other research findings also suggest that lower income groups will be more willing to adopt^55,56^.

Most cotton farmers use their free time to learn about or attend technical training to improve their skills. The local government also organises visits to exhibitions and exchanges of farming experiences^66^. Thus, improving farmers’ education level has a positive impact on their behaviour^67–69^. In addition, with the development of modern information networks, cotton farmers use their smartphones and new media channels such as public websites, academic lectures or the Jitterbug App to obtain more information about SA and search for SA technologies that interest them for in-depth understanding^52,53^. However, there are still some cotton farmers who are affected by factors such as literacy, age, closed production and inability to use smartphone functions proficiently, making it difficult to access the new technologies they need. This suggests that the accessibility of technology and perceived self-efficacy create potential barriers to their behaviour^29,55,70,71^.

## Conclusion and policy recommendations

In order to further optimise agricultural infrastructure, scientific cultivation management and high crop yields and efficiency in the region. Using 394 microscopic research data, this paper uses empirical analysis through DeconstructiveTheory of Planned Behaviour (DTPB) combined with mechanistic analysis to delve deeper into the factors that influence cotton farmers’ adoption of smart agriculture (SA) technologies at the micro level. The findings are as follows: under the behavioural belief dimension, cotton farmers are more interested in the change in welfare level brought about by theSA technology itself. In descending order of intensity, the following factors influence the willingness to adopt: yield, industrial development, labour, economic returns, water quantity and pesticide and fertiliser use; while the higher-than-usual price of technical services for new technologies inhibits their willingness to adopt. The influence of superiors on cotton farmers’ willingness to behave is stronger under the normative belief dimension. Government publicity and subsidies have a greater degree of influence on the willingness to adopt SA technologies, followed by villages and regiments. Dealers, on the other hand, mainly promote agricultural products and not so much new technologies. And in terms of peer influence, the vast majority of cotton farmers listen to the recommendations of their friends and family when making decisions. This is followed by the opinions of family members. Under the control belief dimension, cotton farmers’ willingness to adopt SA technologies is mainly influenced by technological convenience, and information transfer within village groups is more easily accessible than that of online information trading platforms. This is followed by self-efficacy. In addition, cotton farmers believe that only the purchase of machinery costs a lot of money, while the expenditure on technical services is generally acceptable and they will also use their free time to attend various trainings. In addition, behavioural attitudes, subjective norms, and perceived behavioural control can directly contribute to cotton farmers’ willingness and behaviour to adopt SA technologies, and can also indirectly influence behaviour through willingness to adopt. Cotton farmers’ satisfaction with policy and technology has a positive moderating role in the development of SA technology adoption behaviour.

In response to the above findings, the following recommendations are made: First, reduce the cost of adopting SA technology for cotton farmers. The adoption of SA technology services by cotton farmers should be subsidised in accordance with the relevant policies, so as to motivate cotton farmers to change their planting methods and adopt SA technology in the long term. Second, improve the overall level of SA technology. The development of SA is supported by its technology, and the shortcomings and deficiencies of the technology in the actual use of the process need to be remedied as soon as possible. Continuously strengthen the research and development of SA technology, and further improve the integration of SA technology in agricultural production applications. Thirdly, the creation of demonstration references for the adoption of SA technologies. Give full play to the leading role of the government to carry out a number of SA transformation projects based on various modern agricultural demonstration parks and zones. Secondly, constantly stimulate large planters and new business entities to organise models and so on to enthusiastically carry out trial fields of SA technology. Through the display of the actual use of the effect, for other cotton farmers to provide reference basis and demonstration drive, so as to reduce some cotton farmers concerns. In addition, increase the guidance of technical service personnel, the later problems can be timely communication feedback. Fourth, popularise knowledge about SA and expand access to information. Increase the technical training of agriculture-related departments. And use new media platforms such as public numbers, short videos and comprehensive agricultural information service platforms to promote relevant knowledge in multiple directions through various channels such as distance education, expert lectures and online micro-classes. Help cotton farmers more convenient understanding of SA, weaken the disadvantages of information asymmetry of cotton farmers (Supplementary Information).

Research limitations and suggestions for future research. Due to the word limit of the thesis, there are still some elements that have not been explored. From a micro perspective, differences in farmers’ behavioural decisions can be explored in depth through heterogeneity analysis as there are significant differences in individual characteristics and household characteristics of farmers. From a macro perspective, SA technologies in developed countries are relatively mature and commonly used, while developing countries are still in the initial exploration stage. An attempt is made to link them in a comparative analysis to explore what else is preventing the widespread adoption of new technologies in developing countries. This will explore the underlying causes of the gap between developing and developed countries.

## Supplementary Information

Supplementary Information.

## Data Availability

The raw data and collated data supporting the fndings of this study could be made available from the corresponding author upon judicious request.

## References

[1] Pivoto D, Barham B, Waquil PD, Foguesatto CR, Dalla Corte VF, Zhang D, Talamini E (2019). Factors influencing the adoption of smart farming by Brazilian grain farmers. Int. Food Agribus. Manag. Rev..

[2] Kurgat BK, Lamanna C, Kimaro A, Namoi N, Manda L, Rosenstock TS (2020). Adoption of climate-smart agriculture technologies in Tanzania. Front. Sustain. Food Syst..

[3] Pagliacci F, Defrancesco E, Mozzato D, Bortolini L, Pezzuolo A, Pirotti F, Pisani E, Gatto P (2020). Drivers of farmers’ adoption and continuation of climate-smart agricultural practices. A study from northeastern Italy. Sci. Total Environ..

[4] Cheng W, Ma T, Wang X, Wang G (2022). Anomaly detection for internet of things time series data using generative adversarial networks with attention mechanism in smart agriculture. Front. Plant Sci..

[5] Deng F, Jia S, Ye M, Li Z (2022). Coordinated development of high-quality agricultural transformation and technological innovation: A case study of main grain-producing areas, China. Environ. Sci. Pollut. Res..

[6] Bacenetti J, Paleari L, Tartarini S, Vesely FM, Foi M, Movedi E, Ravasi RA, Bellopede V, Durello S, Ceravolo C (2020). May smart technologies reduce the environmental impact of nitrogen fertilization? A case study for paddy rice. Sci. Total Environ..

[7] Ju X (2022). Application of big data technology to promote agricultural structure adjustment and high-quality development of modern agriculture. Comput. Intell. Neurosci..

[8] Li D, Nanseki T, Chomei Y, Kuang J (2022). A review of smart agriculture and production practices in Japanese large-scale rice farming. J. Sci. Food Agric..

[9] Ndiritu SW, Kassie M, Shiferaw B (2014). Are there systematic gender differences in the adoption of sustainable agricultural intensification practices? Evidence from Kenya. Food Policy.

[10] Samoraj M, Mironiuk M, Witek-Krowiak A, Izydorczyk G, Skrzypczak D, Mikula K, Basladynska S, Moustakas K, Chojnacka K (2022). Biochar in environmental friendly fertilizers—Prospects of development products and technologies. Chemosphere.

[11] Adesipo A, Fadeyi O, Kuca K, Krejcar O, Maresova P, Selamat A, Adenola M (2020). Smart and climate-smart agricultural trends as core aspects of smart village functions. Sensors.

[12] Imran MA, Ali A, Culas RJ, Ashfaq M, Baig IA, Nasir S, Hashmi AH (2022). Sustainability and efficiency analysis w.r.t. adoption of climate-smart agriculture (CSA) in Pakistan: A group-wise comparison of adopters and conventional farmers. Environ. Sci. Pollut. Res..

[13] Lim HR, Khoo KS, Chia WY, Chew KW, Ho S-H, Show PL (2022). Smart microalgae farming with internet-of-things for sustainable agriculture. Biotechnol. Adv..

[14] Zhang R, Li X (2021). Edge computing driven data sensing strategy in the entire crop lifecycle for smart agriculture. Sensors.

[15] Mori H, Kundaliya J, Naik K, Shah M (2022). IoT technologies in smart environment: Security issues and future enhancements. Environ. Sci. Pollut. Res..

[16] Huang Y, Chen Z-X, Yu T, Huang X-Z, Gu X-F (2018). Agricultural remote sensing big data: Management and applications. J. Integr. Agric..

[17] Visockiene JS, Tumeliene E, Maliene V (2019). Analysis and identification of abandoned agricultural land using remote sensing methodology. Land Use Pol..

[18] Mignouna HD, Abang MM, Omanya G, Nang’Ayo F, Bokanga M, Boadi R, Muchiri N, Terry E (2008). Delivery of agricultural technology to resource-poor farmers in Africa. Ann. N.Y Acad. Sci..

[19] Kalyani Y, Collier R (2021). A systematic survey on the role of cloud, fog, and edge computing combination in smart agriculture. Sensors.

[20] Bhardwaj A, Kumar M, Alshehri M, Keshta I, Abugabah A, Sharma SK (2022). Smart water management framework for irrigation in agriculture. Environ. Technol..

[21] Jamil I, Jun W, Mughal B, Raza MH, Imran MA, Waheed A (2021). Does the adaptation of climate-smart agricultural practices increase farmers’ resilience to climate change?. Environ. Sci. Pollut. Res..

[22] Lan L, Sain G, Czaplicki S, Guerten N, Shikuku KM, Grosjean G, Laederach P (2018). Farm-level and community aggregate economic impacts of adopting climate smart agricultural practices in three mega environments. PLoS ONE.

[23] Makate C, Makate M, Mango N, Siziba S (2019). Increasing resilience of smallholder farmers to climate change through multiple adoption of proven climate-smart agriculture innovations. Lessons from Southern Africa. J. Environ. Manag..

[24] Gebre GG, Isoda H, Rahut DB, Amekawa Y, Nomura H (2019). Gender differences in the adoption of agricultural technology: The case of improved maize varieties in southern Ethiopia. Women Stud. Int. Forum.

[25] Ngigi MW, Muange EN (2022). Access to climate information services and climate-smart agriculture in Kenya: A gender-based analysis. Clim. Change.

[26] Agbenyo W, Jiang Y, Jia X, Wang J, Ntim-Amo G, Dunya R, Siaw A, Asare I, Twumasi MA (2022). Does the adoption of climate-smart agricultural practices impact farmers’ income? Evidence from Ghana. Int. J. Environ. Res. Public Health.

[27] Wu Y, Ma W (2022). Rural workplace sustainable development of smart rural governance workplace platform for efficient enterprise performances. J. Environ. Public Health.

[28] Barham BL, Chavas J-P, Fitz D, Schechter L (2018). Receptiveness to advice, cognitive ability, and technology adoption. J. Econ. Behav. Organ..

[29] Musafiri CM, Kiboi M, Macharia J, Ng’etich OK, Kosgei DK, Mulianga B, Okoti M, Ngetich FK (2022). Adoption of climate-smart agricultural practices among smallholder farmers in Western Kenya: Do socioeconomic, institutional, and biophysical factors matter?. Heliyon.

[30] Ali E (2021). Farm households’ adoption of climate-smart practices in subsistence agriculture: Evidence from Northern Togo. Environ. Manag..

[31] Wu F (2022). Adoption and income effects of new agricultural technology on family farms in China. PLoS ONE.

[32] Chi L, Han S, Huan M, Li Y, Liu J (2022). Land fragmentation, technology adoption and chemical fertilizer application: Evidence from China. Int. J. Environ. Res. Public Health.

[33] Zhao D, Zhou H (2021). Livelihoods, technological constraints, and low-carbon agricultural technology preferences of farmers: Analytical frameworks of technology adoption and farmer livelihoods. Int. J. Environ. Res. Public Health.

[34] Wang H, Wang X, Sarkar A, Zhang F (2021). How capital endowment and ecological cognition affect environment-friendly technology adoption: A case of apple farmers of Shandong Province, China. Int. J. Environ. Res. Public Health.

[35] Huang B, Kong H, Yu J, Zhang X (2022). A study on the impact of low-carbon technology application in agriculture on the returns of large-scale farmers. Int. J. Environ. Res. Public Health.

[36] Wang L, Tang J, Tang M, Su M, Guo L (2022). Scale of operation, financial support, and agricultural green total factor productivity: Evidence from China. Int. J. Environ. Res. Public Health.

[37] Li K, Li Q (2022). Towards more efficient low-carbon agricultural technology extension in China: Identifying lead smallholder farmers and their behavioral determinants. Environ. Sci. Pollut. Res..

[38] Li Q, Wang J, Wu J, Zhai Q (2022). The dual impacts of specialized agricultural services on pesticide application intensity: Evidence from China. Pest Manag. Sci..

[39] Exposito A, Berbel J (2017). Why is water pricing ineffective for deficit irrigation schemes? A case study in Southern Spain. Water Resour. Manag..

[40] Guo H, Zhao W, Pan C, Qiu G, Xu S, Liu S (2022). Study on the influencing factors of farmers’ adoption of conservation tillage technology in black soil region in China: A logistic-ISM model approach. Int. J. Environ. Res. Public Health.

[41] Gikonyo NW, Busienei JR, Gathiaka JK, Karuku GN (2022). Analysis of household savings and adoption of climate smart agricultural technologies Evidence from smallholder farmers in Nyando Basin, Kenya. Heliyon.

[42] Luo L, Qiao D, Zhang R, Luo C, Fu X, Liu Y (2022). Research on the influence of education of farmers’ cooperatives on the adoption of green prevention and control technologies by members: Evidence from Rural China. Int. J. Environ. Res. Public Health.

[43] Kathage J, Smit B, Janssens B, Haagsma W, Luis Adrados J (2022). How much is policy driving the adoption of cover crops? Evidence from four EU regions. Land Use Pol..

[44] Lopez-Ridaura S, Frelat R, van Wijk MT, Valbuena D, Krupnik TJ, Jat ML (2018). Climate smart agriculture, farm household typologies and food security An ex-ante assessment from Eastern India. Agric. Syst..

[45] Chuang J-H, Wang J-H, Liou Y-C (2020). Farmers’ knowledge, attitude, and adoption of smart agriculture technology in Taiwan. Int. J. Environ. Res. Public Health.

[46] Li W, Ruiz-Menjivar J, Zhang L, Zhang J (2021). Climate change perceptions and the adoption of low-carbon agricultural technologies: Evidence from rice production systems in the Yangtze River Basin. Sci. Total Environ..

[47] Faisal M, Xia C, Akhtar S, Raza MH, Khan MTI, Ajmal MA (2020). Modeling smallholder livestock herders’ intentions to adopt climate smart practices: An extended theory of planned behavior. Environ. Sci. Pollut. Res..

[48] Lee Y-C, Hsieh Y-F, Guo Y-B (2013). Construct DTPB model by using DEMATEL: A study of a university library website. Program-Electron. Libr. Inf. Syst..

[49] Mottaleb KA (2018). Perception and adoption of a new agricultural technology: Evidence from a developing country. Technol. Soc..

[50] Hussain B, Naqvi SAA, Anwar S, Shah SAR, ul Hassan RH, Shah AA (2021). Zig-zag technology adoption behavior among brick kiln owners in Pakistan. Environ. Sci. Pollut. Res..

[51] Zhou W, Qing C, Deng X, Song J, Xu D (2022). How does Internet use affect farmers’ low-carbon agricultural technologies in southern China?. Environ. Sci. Pollut. Res..

[52] Li B, Zhuo N, Ji C, Zhu Q (2022). Influence of smartphone-based digital extension service on farmers’ sustainable agricultural technology adoption in China. Int. J. Environ. Res. Public Health.

[53] Yao S, Wu G (2022). Research on the efficiency of green agricultural science and technology innovation resource allocation based on a three-stage DEA model—A case study of Anhui Province, China. Int. J. Environ. Res. Public Health.

[54] Buehren N, Goldstein M, Molina E, Vaillant J (2019). The impact of strengthening agricultural extension services on women farmers: Evidence from Ethiopia. Agric. Econ..

[55] Cofe O, Adeoti A, Nkansah-Boadu F, Awuah E (2010). Farmers perception and economic benefts of excreta use in southern Ghana. Resour. Conserv. Recycl..

[56] Dansol G (2002). Farmers’ perception and willingness to pay for urban waste compost in Ghana. Waste Manag. Environ..

[57] Hu H, Hu H, Cao A, Chen S, Li H (2022). Effects of risk perception of pests and diseases on tea famers’ green control techniques adoption. Int. J. Environ. Res. Public Health.

[58] Rouse J, Rothenberger S, Zurbrügg C (2008). Marketing Compost A Guide for Compost Producers in Low and Middle-Income Countries.

[59] Burlakovs J (2017). Paradigms on landfll mining: From dump site scavenging to ecosystem services revitalization. Resour. Conserv. Recycl..

[60] Cooley L, Howard J (2019). Scale Up Sourcebook.

[61] Ren Z, Fu Z, Zhong K (2022). The influence of social capital on farmers’ green control technology adoption behavior. Front. Psychol..

[62] Wang G, Lu Q, Capareda SC (2020). Social network and extension service in farmers’ agricultural technology adoption efficiency. PLoS ONE.

[63] Mugivhisa LL, Olowoyo JO (2015). An assessment of university students and staf perceptions regarding the use of human urine as a valuable soil nutrient in South Africa. Afr. Health Sci..

[64] Wilde BC, Lieberherr E, Okem AE, Six J (2019). Nitrifed human urine as a sustainable and socially acceptable fertilizer: An analysis of consumer acceptance in Msunduzi, South Africa. Sustainability..

[65] Gwara S, Wale E, Odindo A (2022). Behavioral intentions of rural farmers to recycle human excreta in agriculture. Sci. Rep..

[66] Kohl, R. & Foy, C. *Guide to the Agricultural Scalability Assessment Tool for Assessing and Improving the Scaling Potential of Agricultural Technologies* (2018).

[67] Phuc PD, Konradsen F, Phuong PT, Cam PD, Dalsgaards A (2006). Practice of using human exceta as fertilizer and implications for health in Nghean Province, Vietnam. Southeast Asian. J. Trop. Med. Public Health.

[68] Mariwah S, Drangert J-OO (2011). Community perceptions of human excreta as fertilizer in peri-urban agriculture in Ghana. Waste Manag. Res..

[69] Mugivhisa LL, Olowoyo JO, Mzimba D (2017). Perceptions on organic farming and selected organic fertilizers by subsistence farmers in Ga-Rankuwa, Pretoria, South Africa. Afr. J. Sci. Technol. Innov. Dev..

[70] Andersson E (2015). Turning waste into value: Using human urine to enrich soils for sustainable food production in Uganda. J. Clean. Prod..

[71] Lagerkvist CJ, Shikuku K, Okello J, Karanja N, Ackello-Ogutu C (2015). A conceptual approach for measuring farmers’ attitudes to integrated soil fertility management in Kenya. NJAS Wageningen J. Life Sci..

